# Study of Malformin C, a Fungal Source Cyclic Pentapeptide, as an Anti-Cancer Drug

**DOI:** 10.1371/journal.pone.0140069

**Published:** 2015-11-05

**Authors:** Jing Wang, Zaoli Jiang, Wing Lam, Elizabeth A. Gullen, Zhe Yu, Ying Wei, Lihui Wang, Caroline Zeiss, Amanda Beck, Ee-Chun Cheng, Chunfu Wu, Yung-Chi Cheng, Yixuan Zhang

**Affiliations:** 1 Department of Pharmacology, Yale University School of Medicine, New Haven, Connecticut, United States of America; 2 School of Life Science and Biopharmaceutics, Shenyang Pharmaceutical University, Shenyang, China; 3 Section of Comparative Medicine, Yale University School of Medicine, New Haven, Connecticut, United States of America; 4 Department of Oncology and Hematology, Dongzhimen Hospital Affiliated to Beijing University of Chinese Medicine, Beijing, China; Winship Cancer Institute of Emory University, UNITED STATES

## Abstract

Malformin C, a fungal cyclic pentapeptide, has been claimed to have anti-cancer potential, but no *in vivo* study was available to substantiate this property. Therefore, we conducted *in vitro* and *in vivo* experiments to investigate its anti-cancer effects and toxicity. Our studies showed Malformin C inhibited Colon 38 and HCT 116 cell growth dose-dependently with an IC_50_ of 0.27±0.07μM and 0.18±0.023μM respectively. This inhibition was explicated by Malformin C’s effect on G2/M arrest. Moreover, we observed up-regulated expression of phospho-histone H2A.X, p53, cleaved CASPASE 3 and LC3 after Malformin C treatment, while the apoptosis assay indicated an increased population of necrotic and late apoptotic cells. *In vivo*, the pathological study exhibited the acute toxicity of Malformin C at lethal dosage in BDF1 mice might be caused by an acute yet subtle inflammatory response, consistent with elevated IL-6 in the plasma cytokine assay. Further anti-tumor and toxicity experiments proved that 0.3mg/kg injected weekly was the best therapeutic dosage of Malformin C in Colon 38 xenografted BDF1 mice, whereas 0.1mg/kg every other day showed no effect with higher resistance, and 0.9mg/kg per week either led to fatal toxicity in seven-week old mice or displayed no advantage over 0.3mg/kg group in nine-week old mice. Overall, we conclude that Malformin C arrests Colon 38 cells in G2/M phase and induces multiple forms of cell death through necrosis, apoptosis and autophagy. Malformin C has potent cell growth inhibition activity, but the therapeutic index is too low to be an anti-cancer drug.

## Introduction

**Fig 1:**
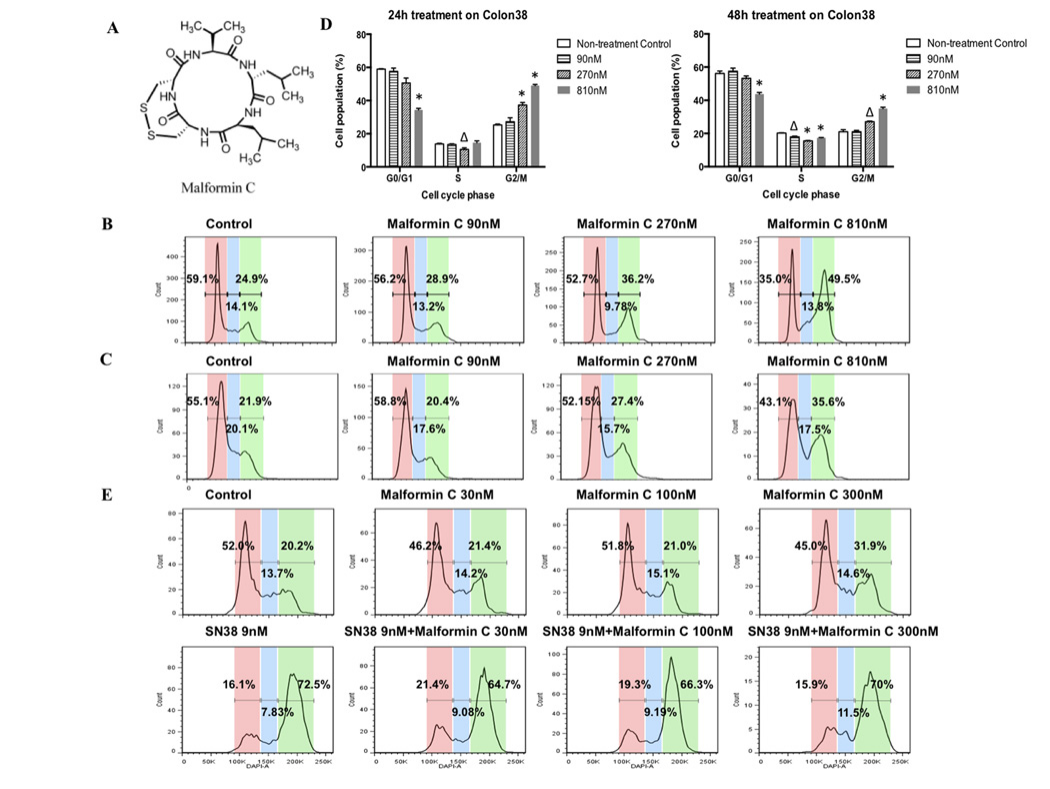
Cell cycle analysis of Colon 38 cells treated by Malformin C and its combinations. **(A)** Chemical structure of Malformin C. Malformin C is a member of Malformins, a group of fungal cyclic pentapeptides. Its chemical formula is C_23_H_39_O_5_N_5_S_2_ with a molecular weight of 529.7. **(B)** Cell cycle progression of Colon 38 cells exposed to an increasing concentration of Malformin C for 24 hours. **(C)** Cell cycle progression of Colon 38 cells exposed to an increasing concentration of Malformin C for 48 hours. **(D)** The dose-dependent accumulation of G2-M phase Colon 38 cells treated by Malformin C at concentrations of 90 nM, 270 nM and 810 nM. Compared to the control group, the symbol Δ represented *P*<0.05, while * represented *P*<0.01. **(E)** Cell cycle analysis of Colon 38 cells treated with combinations of Malformin C and SN38. All the cells were exposed to respective compounds indicated above the graph for 24 hours and the analysis was done in duplicate. When treated with SN38, no significant changes of cell cycle progression were observed without or with different dosage of Malformin C.

Malformins are a group of cyclic pentapeptides originally discovered and isolated from culture filtrate of the fungus *Aspergillus niger*, and it induces the malformations of bean plants and curvatures of corn roots [1, 2]. Malformin could also be obtained from the extract of *Aspergillus tubingensis* [3]. At present, three sub-groups of Malformins are identified, namely Malformin A, Malformin B [4], and Malformin C. As the first discovered sub-group, Malformin A mainly consists of Malformin A_1_, A_2_, A_3_ and A_4_ [5, 6], in which Malformin A_1_ is most well-studied, and its biological activities have been reported including malformations of plants, antibiotic effects against certain bacteria species [7], enhancement of fibrinolytic activity [8, 9], and prevention against IL1-induced procoagulant reaction [10]. Malformin C is a relatively new and toxic member of Malformins [11] (Fig 1A). It has shown antibacterial activity [12], as well as potent antimalarial and antitrypanosomal properties [13]. Also, Malformin C inhibits bleomycin-induced G2 checkpoint in Jurkat cells [14], and was claimed to have potential in cancer treatment. However, no *in vivo* study has been presented to substantiate its anti-tumor property. Therefore, we carried out a series of preliminary *in vitro* and *in vivo* studies to explore Malformin C’s anti-cancer effects and its *in vivo* toxicity.

Although the incidence and mortality of colon and rectal cancers have decreased in the past twenty years, colorectal cancer (CRC) remains the fourth most frequently diagnosed cancer and the second leading cause of cancer death in the United States [15]. Chemotherapy is commonly used for CRC at different stages. Irinotecan (CPT-11, Camptosar) is a chemotherapeutic drug that prevents DNA from unwinding by inhibition of topoisomerase I action, and is used for the treatment of advanced or metastatic CRC. In this study, we used murine Colon 38 cells, HCT116 cells and allograft tumor as the model to study the potential of Malformin C’s anti-cancer property and chose CPT-11 as a control to explore its possible combination and underlying mechanism.

## Materials and Methods

### Fungal Material

The culture of *Aspergillus tubingensis* was isolated from the sand soil of the Patong beach of Phuket, Thai island (7.89°N, 98.29°E). The sand soil was collected from a public beach, and no specific permission was required for that location or such activity. We confirm that our field studies did not involve endangered or protected species. The isolate was identified by one of the authors (Prof. Yixuan Zhang) based on sequence analysis of the ITS region of the rDNA and its morphology. The strain was assigned the Accession No. 6992 culture collection at the China General Microbiological Culture Collection Center, Beijing. The fungal strain was cultured on slants of potato dextrose agar (PDA) at 28°C for 4 days. Then the slants spores were inoculated in 250 ml Erlenmeyer flasks, each containing 40ml of media (1% glucose, 1% sucrose, 1% peptone, 0.5% peanut meal, 0.3% KH_2_PO_4_, 0.1% NH_4_Cl, 0.3% MgSO_4_·7H_2_O) and the final pH of the media was adjusted to 6.5 before sterilization. Flask cultures were incubated at 28°C on a rotary shaker at 160 rpm for 48 hours to obtain seed liquid for fermentation. Fermentation was carried out in Fernbach culture flasks (500ml) each containing 80ml of media (1% glucose, 0.2% aspartic acid, 0.1% KH_2_PO_4_, 0.05% MgSO_4_·7H_2_O and trace elements) and the final pH 6.0 before sterilization. The trace elements per 1L fermentation media contained 0.1mg MnSO_4_, 0.1mg ZnSO_4_, 0.2mg FeCl_3_, 1mg Vitamin B_1_, 5μg biotin. 8% (volume ratio) seed liquid were planted into each fermentation bottle, then cultured at 28°C on a rotary shaker at 160 rpm for 96 hours.

### Extraction, isolation and identification of Malformin C

20L fermentation material was vacuum filtered to remove mycelium, then extracted with N-butyl alcohol, and the organic solvent was evaporated to dryness under vacuum to yield a crude extract. 5g crude extract was dissolved in CH_3_OH followed by silica gel column chromatography (CC) (10×100cm) using CHCl_3_-CH_3_OH gradient elution (15:1→10:1→5:1). The fraction (0.8g) eluted with 15:1 CHCl_3_-CH_3_OH was separated by column chromatography, and further purification by RP-HPLC (Shimadzu LC-10A; Diamonsil C18 200×4.6mm 5μm; 1ml/min, 80% CH_3_OH in H_2_O for elution, detection UV wavelength at 215nm, malformin C t_R_ 13.065min) to 99% purity. The pure compound was identified on the basis of extensive spectroscopic investigation including HR-ESI-MS, 1D-NMR and 2D-NMR. HR-ESI-MS spectra were obtained on a Bruker micro-Q-TOF mass spectrometer. 1D and 2D-NMR spectra were measured on a Bruker ARX-300 spectrometer (300 MHz for ^1^H, 75 MHz for ^13^C). The pure compound was identified as malformin C via comparison of data with those previously reported [16].

### Drugs and Cell Lines

CPT, doxorubicin, vincristine, Taxol, VP-16, Oxaliplatin and DAPI were purchased from Sigma. L-OddC was provided by Shire Pharmaceuticals, Inc. Murine Colon 38 cell line was established before [17] and obtained from Dr. Giuseppe Pizzorno of Translational Science in Nevada Cancer Institute, USA. Human lung adenocarcinoma NCI-H1975 cell line was purchased from National Cancer Institute and given by Dr. Don Nguyen of the department of pathology at Yale University. Human lymphoid CEM, human nasopharyngeal carcinoma KB and HCT 116 colon cancer cell lines were purchased from American Type Culture Collection (ATCC). Murine pancreatic cancer PanO2 and KB-resistant cell lines were previously established in the lab and described before [18–22]. Colon 38, NCI-H1975, CEM and KB cells were cultured in RPMI 1640 medium, PanO2 cells were cultured in DMEM medium, and HCT 116 cells in McCoy's 5a medium. KB-resistant cell lines were cultured in RPMI 1640 with 37nmol/L Doxorubicin for KB-MDR, 300nM CPT for KB300-CPT, 20nM Vincristine for KBv20c, 20μM VP16 for KB20a-VP16 and 7μM VP16 for KB7d, and all those drugs were withdrawn from the media once cells were seeded for the experiments. All cells were cultured in media supplemented with 10% FBS, and maintained at 37°C in a humidified atmosphere of 5% CO_2_.

### Cell Growth Assay

Cells were seeded at 1×10^4^/ml in 48-well plates. After overnight incubation, Colon 38, HCT 116, PanO2, NCI-H1975, CEM, KB, KB-MDR, KB300-CPT, KBv20c, KB20aVP16, and KB7d cells were treated with drugs for 72 hours, 72 hours, 72 hours, 120 hours, 72 hours, 120 hours, 72 hours, 108 hours, 72 hours, 108 hours, and 108 hours, respectively. Cells were fixed and stained with 0.5% methylene blue in 50% ethanol for 2 hours at room temperature (RT), followed by washing with tap water to remove the unabsorbed methylene blue. Plates were air dried overnight, solubilized in 1% Sarkosyl and rotated for 3 hours at room RT. Cell growth was quantitated based on the amount of methylene blue adsorbed to interact with cellular proteins measured by spectrophotometer (Molecular Devices) at 595 nm. The values were averaged and the day zero value was subtracted from each averaged value including the untreated control. The drug treated values were expressed as a percentage of the untreated control, and percentages were plotted vs. drug concentration to generate an IC_50_ value. All experiments were performed in duplicate wells and were repeated at least three times.

### Clonogenic Assay

Colon 38 and HCT 116 cells were plated at 2.4×10^5^/well in six-well plates respectively and treated with Malformin C or Oxaliplatin for 24 hours. The next day cells were harvested and seeded into new six-well plates at 300/well with fresh culture medium. The colonies were stained with methylene blue after 11 days and then counted. The results included the means and S.D. obtained from three independent experiments.

### Cell Cycle Analysis

Colon 38 and HCT 116 cells were seeded separately onto six-well plates at 3×10^5^ cells per well, and incubated for 48 hours. Various concentrations of Malformin C or Oxaliplatin were added to the culture media and incubated for another 24 hours. Cells were then harvested using pancreatin and 10μg/mL DNase1, 37°C, 5min and washed twice with cold PBS. A total of 1x10^6^ cells for each sample were aliquoted, resuspended in 500μL PBS and fixed by adding 500μl 4% PFA. After 30 minutes incubation on ice, the samples were washed twice with cold PBS. Prior to analysis cells were resuspended in 150μL 1ug/ml DAPI in PBS for 30 minutes, then filtered to remove aggregates. Analysis was carried out on a BD Biosciences LSRII and analyzed using FlowJo software (Tree Star).

### Apoptosis Assay

Early apoptotic events were determined by using a Vybrant Apoptosis Assay Kit #2 (Molecular Probes, V13241) according to the manufacturer’s instructions, and the method described before [23]. Colon 38 cells were treated with different concentrations of Malformin C and CPT for 24 hours. Cells treated with 3.7% formaldehyde for 15 minutes were used as positive control cells. Analysis was carried out on a BD Biosciences LSRII and analyzed using FlowJo software (Tree Star).

### Confocal Microscopy

Colon 38 cells were treated with different concentrations of Malformin C for three different time courses including 24-hour treatment, 48-hour treatment, and 24-hour treatment followed by Malformin C withdrawal and another 24-hour incubation. All the cells were fixed with 4% paraformaldehyde in PBS and then permeabilized with 0.5% Triton X-100 in PBS. 3% bovine serum albumin in PBS was used to block nonspecific binding. Cells were then exposed to monoclonal anti-p53, monoclonal anti-cleaved CASPASE 3, or monoclonal anti-LC3 (1:200; Cell Signaling Technology) at RT for 1 hour, washed with 0.2% Tween20 in PBS and followed by fluorescein isothiocyanate-conjugated anti-rabbit IgG at 1:400 dilution. Cytoplasmic actin was counter-stained with 0.25μg/ml rhodamine phalloidin (Invitrogen). The cells were then sealed in antifade reagent (Invitrogen). Confocal micrographs were scanned by a laser scanning confocal microscope (LSM 510; Carl Zeiss, Inc., Thornwood, NY).

### Western Blot

Colon 38 cells and HCT 116 (5×10^5^) were plated onto 6-well plates. After 24 hours, cells were treated as indicated in the figure legends. Cells were lysed in 2×SDS sample buffer (26.7mM pH 6.8 Tris-HCl, 1% SDS, 25% glycerol, 0.36M β-mercaptoethanol, and 0.05% bromphenol blue) and sonicated for 10 seconds to shear DNA. The whole-cell extracts were then electrophoresed through 15% or 8% SDS-polyacrylamide gels and transferred to nitrocellulose membranes (Bio-rad). They were then incubated for 1 hour at RT with blocking solution (TBS, 0.1% Tween 20, and 3% nonfat milk), followed by a specific antibody to phospho-Histone H2A.X (Ser139) (1:2000, rabbit monoclonal mAb; Cell Signaling Technology, #9718), total H2A.X (1:2000, rabbit monoclonal mAb; Cell Signaling Technology, #7631), CASPASE 3 (D2R6Y) (1:1000, rabbit monoclonal mAb; Cell Signaling Technology, #14220), and LC3A (D50G8) (1:1000, rabbit monoclonal mAb; Cell Signaling Technology, #4599) overnight at 4°C. The membranes were then further incubated with horseradish peroxidase-conjugated anti-rabbit IgG (1:2000; Sigma) at RT for 2 hours, and signals were visualized by enhanced chemiluminescence (Perkin-Elmer Life Science). The phospho-Histone H2A.X and total H2A.X blots were reprobed with antibodies to β-Actin (1:2000; Sigma, A5316).

### Animal Studies

A total of thirty 4±0.5 week-old female BDF1 mice (Charles River Laboratories) were used for the first experiment, twenty-five 6±0.5 week-old mice for the second experiment, and six 6±0.5 week-old mice for pathological study. All the mice were housed for two weeks prior to the experiment to adjust to the environment at the Yale Animal Facility, specific pathogen free (SPF), 23±2°C, 12:12 light and dark cycle, 5 per cage. 2×10^6^ murine Colon 38 cells were transplanted subcutaneously into each mouse for the experiment. We daily monitored the weight of the animal, loss of mobility, decreased body temperature and abnormal movement or posture, lack of grooming activity, dehydration as judged by A 2–3 sec tent time and/or if the mouse lost greater than 15% body weight, it would be removed from the study. Any animal that appeared very sick that was cold to the touch, hunched, could not move, eat or drink normally from the treatment was euthanized with 30% isoflurane (mixture of 30% v/v isoflurane and 70% propylene glycol) inhalation and cervical dislocation. After 10 to 14 days, mice with tumor sizes of 150–300 mm^3^ were selected into a pool, and then randomly allocated to different groups, with 5 mice in each group. Malformin C was dissolved at 10mM DMSO and the final concentration of DMSO was lower than 0.05% of the Malformin C solution used for treatment. Different concentrations of sterilized malformin C (0.1mg/kg, 0.3mg/kg, 0.9mg/kg, 1.8mg/kg, 2.6mg/kg) and sterilized PBS were administered intra-peritoneally (i.p.) in the morning from Day 1. After the whole treatment, blood samples were collected following anesthetization with 30% isoflurane inhalation, and then the mice were euthanized by cervical dislocation. Tumor tissue samples were collected after the mice were confirmed dead and frozen in -70°C for future study. For pathological study of Malformin C’s acute toxicity, we randomly assigned six mice into PBS group and 1.8mg/kg Malformin C group. Drugs were injected i.p. and all the subjects were monitored every 15 minutes for 6 hours until mice in Malformin C group were close to death. Then mice were euthanized, blood samples were taken for chemistry, part of the spleen and peritoneum were cultured for bacteriology, and the rest of tissues examined were fixed in formalin, embedded in paraffin, and sectioned for histopathologic studies. All animal experiments were carried out in accordance with an approved Yale University Institutional Animal Care and Use Committee (IACUC) protocol (2013–07784). All efforts were made to minimize suffering. All sections of this report adhere to the ARRIVE Guidelines for reporting animal research.

### Plasma cytokine assay

The plasma cytokine assay was carried out following the instruction manual of BD Cytometric Bead Array (CBA) Mouse Th1/Th2 Cytokine Kit instruction manual. The mouse Th1/Th2 cytokine standards were reconstituted in assay diluent and serially diluted to the concentration of 0-5000pg/ml. An amount of 10μl of each test mouse cytokine capture bead suspension was mixed, and 50μl of standard dilutions or test mixed beads were transferred to assay tubes. After adding 50μl of PE detection reagent, the samples were incubated at RT for 2 hours. Then the samples were washed, and 300μl of wash buffer was added to each tube. All the samples were analyzed using BD FACS Array System.

### Statistical Analysis

Data was analyzed by one- or two-way analysis of variance (ANOVA) (GraphPad Prism 5), Student’s t test (Microsoft Office Excel), and correlation analysis (GraphPad Prism 5). The difference was considered to be statistically significant when *P*<0.05.

## Results

### The growth inhibition and clonogenicity of Malformin C against different cancer cell lines

The effects of Malformin C on the growth inhibition of different cancer cell lines were examined. All the cells were exposed to increasing concentrations of Malformin C and L-OddC. The IC_50_ value was defined as the concentration of drug that inhibited cell growth by 50%. L-OddC, also known as troxacitabine, is a L-nucleoside analogue with anticancer activity [24–27] and was used as a positive control in this study. Malformin C inhibited Colon 38, HCT 116, PanO2, NCI-H1975, CEM and KB cells in a dose-dependent manner with an IC_50_ of 0.27±0.07 μM, 0.18±0.02 μM, 0.29±0.05 μM, 0.16±0.04 μM, 0.030±0.008 μM and 0.18±0.05 μM respectively (S1 Table). Malformin C had different inhibition effects among different cancer cell lines (*P*<0.01, one-way ANOVA), and the Malformin C was more potent than L-OddC for growth inhibition of Colon 38, HCT 116 and PanO2 (*P*<0.01, t test). Therefore, we selected Colon 38 and HCT 116 for the following study. The actions of Malformin C on the colony-forming ability of Colon 38 and HCT116 were then determined as the percentage of visible colony numbers of drug-treated group compared to non-treatment control group by clonogenic assay. Colon 38 and HCT 116 cells exhibited a dose-dependent loss of colony-forming ability after exposed to Malformin C with a LC_50_ value of 0.6±0.07 μM and 0.7±0.09 μM respectively, which was defined as the concentration of drug that inhibited colony formation by 50%. The ratio of LC_50_ to IC_50_ was approximately 2:1 in Colon 38 cells and 4:1 in HCT 116 cells, and this result indicated that the Malformin C’s effects on the growth inhibition of Colon 38 and HCT 116 were partially irreversible.

### Effects of Malformin C on anticancer drug resistant cell lines

The cross-resistance profiles of Malformin C, in comparison with other conventional anti-cancer drugs, were studied employing KB cell line and a number of well-characterized KB drug-resistant cell lines (Table 1). Malformin C displayed a 233-fold resistance to KB-MDR cells that overexpressed human P-gp 170 protein, when compared to KB cells (*P*<0.0001). It was also 4.4-fold resistant to KBv20c cells (*P*<0.001) and 2.8-fold more sensitive to KB300-CPT cells (*P*<0.05) compared to KB cells (Table 2). In order to further confirm Malformin C’s resistance to KB-MDR cells, Verapamil (VRP), a calcium channel antagonist of P-gp 170 protein, was used to reverse multidrug resistance [28]. A non-toxic concentration of VRP (5 μM) was administrated to KB resistant cells in addition to Malformin C, Taxol and Doxarubicin for 72 hours. With VRP, the IC_50_ of Malformin C for KB-MDR had significantly decreased, from 233-fold to 4-fold of that of KB cells (Table 3). This data shows that KB-MDR cells were highly resistant to Malformin C and the drug-resistant effect was reversed by VRP.

**Table 1:** Characteristics of different KB resistant cell lines.

KB resistant cells	Biological changes	Resistant to
KB-MDR	P-glycoprotein ↑	VP-16, Taxol, Adriamycin, Vincristine [21], [28]
KB300-CPT	Topo I ↓	CPT [21]
KBv20c	P-glycoprotein ↑	VCR [18]
KB20aVP16	Topo II↓,	VP-16, Vincristine, Adriamycin, Doxorubicin [19]
KB7d	Topo II↓, MRP↑	VP-16, Vincristine, Adriamycin, Doxorubicin [19], [20]

**Table 2:** Growth inhibition of KB and its drug-resistant cells by Malformin C and anti-cancer drugs.

	IC_50_ (μM)					
KB cell lines	KB	KB-MDR	KB300-CPT	KBv20c	KB20aVP16	KB7d
Malformin C	0.18±0.05	42±6	0.065±0.005	0.79±0.07	0.12±0.01	0.23±0.05
CPT	0.0032±0.0003	0.0036±0.0002	0.27±0.06	0.0032±0.0003	0.0025±0.0002	0.0030±0.0006
Vincristine	0.020±0.005	1.5±0.2	0.025±0.005	0.5±0.08	0.47±0.07	0.28±0.04
VP16	0.34±0.1	9.6±1.4	0.16±0.01	0.75±0.06	70±3.7	65±5
Taxol	0.048±0.007	3.0±0.3	0.13±0.02	0.024±0.001	0.082±0.003	0.024±0.001
Doxarubicin	0.012±0.002	0.56±0.1	0.013±0.002	0.044±0.01	1.16±0.05	1.5±0.2
L-OddC	0.34±0.1	0.32±0.06	2.1±0.6	0.21±0.001	0.149±0.09	0.54±0.08

Note: Values were means ± SD from more than three independent experiments, with each data point done in duplicate, and all the cells were exposed to different drugs for 72 hours. MDR, multidrug resistance; MRP, multidrug resistance protein; CPT, camptothecin; VP-16, etoposide; Topo I, topoisomerase I; Topo II, topoisomerase II. Malformin C was 233-fold resistant to KB-MDR cells than KB cells (P<0.0001).

**Table 3:** Multidrug resistance of Malformin C and reversal effects of Verapamil.

	IC_50_ (μM)			Folds of KB	
Drug/Compound	KB	KB-MDR	KB-MDR (+VRP)	KB-MDR	(+VRP)
Malformin C	0.18±0.05	42±5	0.76±0.053	233	4
Taxol	0.048±0.007	3.0±0.3	0.20±0.013	63	4
Doxarubicin	0.012±0.002	0.56±0.1	0.033±0.012	47	3

Note: Values were means ± SD from more than three independent experiments, with each data point done in duplicate, and all the cells were exposed to different drugs for 72 hours. Verapamil (VRP) was added to the media at a concentration of 5 μM. VRP’s IC_50_ of KB cells were 34±1 μM, and no toxicity was observed at 5 μM.

### Malformin C caused G2/M arrest in Colon 38 cell line

The impacts of Malformin C on cell cycle phase distribution of Colon 38 and HCT 116 cells were examined and a concentration of 90nM, 270nM, 810nM of Malformin C was administrated respectively for 24 hours and 48 hours. As illustrated in Fig 1, the percentage of G2-M phase gradually yet significantly increased in Colon 38 cells treated with 270nM and 810nM Malformin C in both 24-hour (Fig 1B and 1D) and 48-hour (Fig 1C and 1D) experiments. Conversely, the percentage of G1 phase decreased in cells exposed to 810nM Malformin C. These results implied that Malformin C induced a dose-dependent G2-M arrest in Colon 38 cells. However, no time-dependent changes of cell cycle progression were observed in Colon 38 cells (Fig 1D, S1 Fig), and no cell cycle pattern changes were found in HCT 116 cells (S2 Fig). Furthermore, we examined the effects of Malformin C on the cell cycle progression of Colon 38 cells exposed to SN38 for 24 hours. All the cells were treated with an increasing dosage of Malformin C (0nM, 30nM, 100nM, 300nM) in addition to no drug or 9nM SN38, respectively. Irinotecan (CPT-11) is an analog of camptothecin-a topoisomerase I inhibitor. Inside the body, it is converted into the active metabolite SN38 (7-ethyl-10-hydroxy-camptothecin) by hepatic and intestinal carboxyesterases, and SN38 has a much higher potency *in vitro* causing serious DNA damage which leads to G2-M arrest in cancer cells. In this study, again we confirmed that when treated with 300nM Malformin C alone, the percentage of G2-M phase Colon 38 cells increased significantly compared to the control (Fig 1E). Additionally, SN38 induced G2-M arrest in Colon 38 cells and served as a positive control. There was no significant difference of the progression pattern between Colon 38 cells treated with SN38 alone and those treated with Malformin C in addition to SN38 (Fig 1E).

### Irreversible action of Malformin C in causing cancer cell death

**Fig 2:**
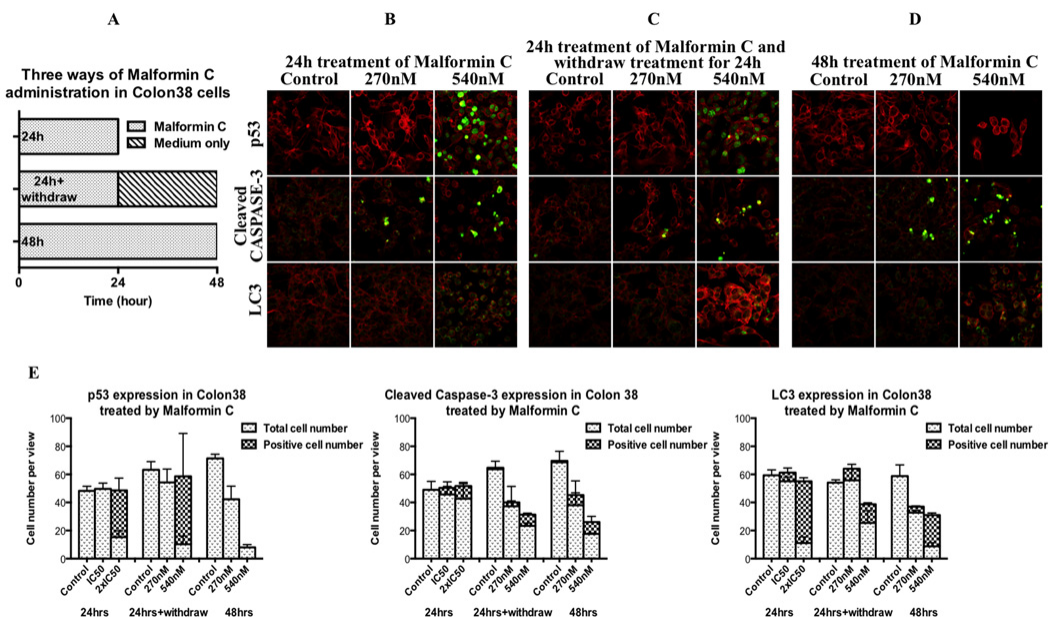
Immunofluorescence micrographs of the p53, cleaved CASPASE 3 and LC3 in Malformin C treated Colon 38 cells. The expression level (green fluorescence) was probed with specific monoclonal p53, cleaved CASPASE 3 and LC3 antibodies respectively, followed by FITC-conjugated anti-mouse IgG. Cytoplasmic Actin (red fluorescence) was counterstained with rhodamine phalloidin. Immunofluorescence micrographs are taken by confocal microscope. **(A)** Experiment design-Malformin C was given for 24h and stained at 24h, for 24h and stained at 48h, for 48h and stained at 48h, respectively. **(B)** Immunofluorescence staining at 24h after 24h treatment. **(C)** Immunofluorescence staining at 48h after 24h treatment. **(D)** Immunofluorescence staining at 48h after 48h treatment. **(E)** Expression of p53, cleaved CASPASE 3 and LC3 after three ways of administration of different concentration of Malformin C based on confocal microscopy results.

Induction of tumor suppressor protein p53 is a key event for cells in response to DNA damage [29]. Most of chemotherapeutic agents for cancer treatment work through DNA damage, resulting in cell death by apoptosis, autophapy or necrosis [30]. For apoptosis, both extrinsic and intrinsic pathways lead to *CASPASE 3* activation which eventually induces apoptosis, and therefore cleaved CASPASE 3 can be used to evaluate the apoptosis process [31]. For autophagy, detection of microtubule-associated protein light chain 3 (LC3) is widely used to monitor autophagy and autophagy-related processes, including autophagic cell death [32]. In this study, p53, cleaved CASPASE 3 and LC3 were examined applying confocal microscope. All the Colon 38 cells for confocal were treated with Malformin C at the concentrations of 0 μM, 0.27 μM and 0.54 μM for 24 hours, 24 hours followed by Malformin C withdrawal and another 24 hours culture by changing media, and 48 hours, respectively (Fig 2A). The expression and location of p53 was shown in immunofluorescence micrographs that p53 expression was induced in the nucleus after exposed to 0.54 μM Malformin C for 24 hours regardless whether cells were cultured for another 24 hours or not (Fig 2B–2D). When treated for 48 hours, although p53 was not shown in cells, the cell number was significantly reduced, which indicated those cells expressing p53 were probably dead from DNA damage (Fig 2E). Also, the expressions of cleaved CASPASE 3 and LC3 were higher in Malformin C treated Colon 38 cells, especially at the concentration of 0.54 μM, which signified there were more cells experiencing apoptosis and autophagy after being exposed to Malformin C (Fig 2B–2E).

### Malformin C led to multiple forms of cell death

**Fig 3:**
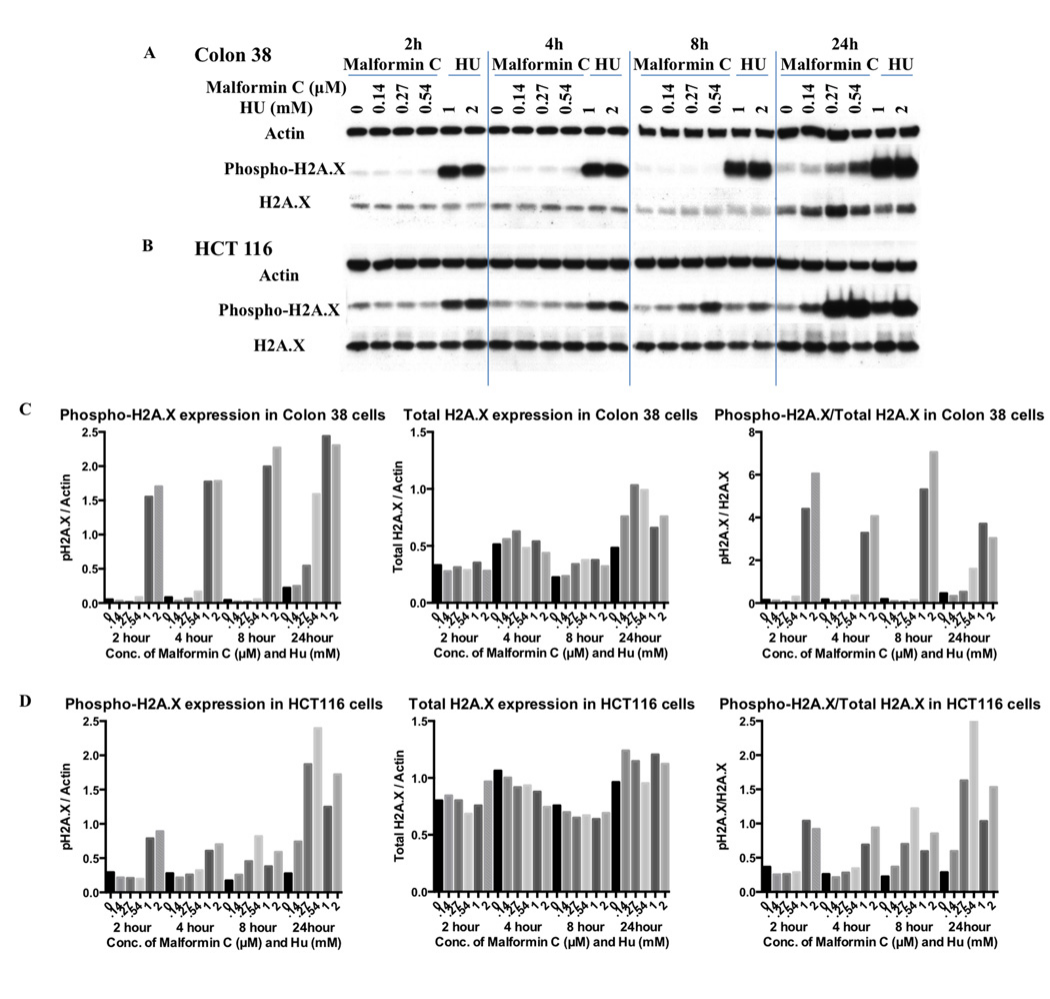
Expression of phosphorylated and total H2A.X in Colon 38 and HCT 116 cells treated with Malformin C. **(A, C)** The expression of phosphorylated and total H2A.X in Colon 38 cells treated with different concentrations of Malformin C (0μM, 0.14μM, 0.27μM, 0.54μM) and Hydroxyurea (1mM, 2mM) for 2-hour, 4 hours, 8 hours and 24 hours tested by Western blot, with β-Actin expression as an internal control. Hydroxyurea was used as positive control. H2A.X is phosphorylated at Ser139. **(B, D)** The expression of phosphorylated and total H2A.X in HCT116 cells treated with different concentrations of Malformin C (0μM, 0.14μM, 0.27μM, 0.54μM) and Hydroxyurea (1mM, 2mM) for 2 hours, 4 hours, 8 hours and 24 hours tested by Western blot, with β-Actin expression as an internal control. Hydroxyurea was used as positive control. H2A.X is phosphorylated at Ser139.

Histone H2A.X is a variant histone that represents approximately 10% of the total H2A histone proteins in normal human fibroblasts. Within minutes following DNA damage, H2A.X is phosphorylated at Ser139 at sites of DNA damage [33]. In our study, Colon 38 cells and HCT 116 cells were treated with Malformin C for 2 hours, 4 hours, 8 hours and 24 hours, and the expressions of phospho-Histone H2A.X and total H2A.X were examined by Western blot. As shown in Fig 3, there was a dose-dependent up-regulated expression of phosphorylated H2A.X at Ser139 after 24-hour treatment of Malformin C in both Colon 38 and HCT 116 cells, while the expression of total H2A.X increased only in Colon 38 cells after 24-hour treatment of Malformin C. Also, a minor dose-dependent increase of phospho-H2A.X expression was observed after 8-hour treatment of Malformin C in HCT 116 cells. When we took the ratio between phosphorylated and total H2A.X, it indicated that Malformin C caused the phosphorylation of H2A.X in HCT 116 cells in a dose-dependent manner when treated for 8–24 hours.

**Fig 4:**
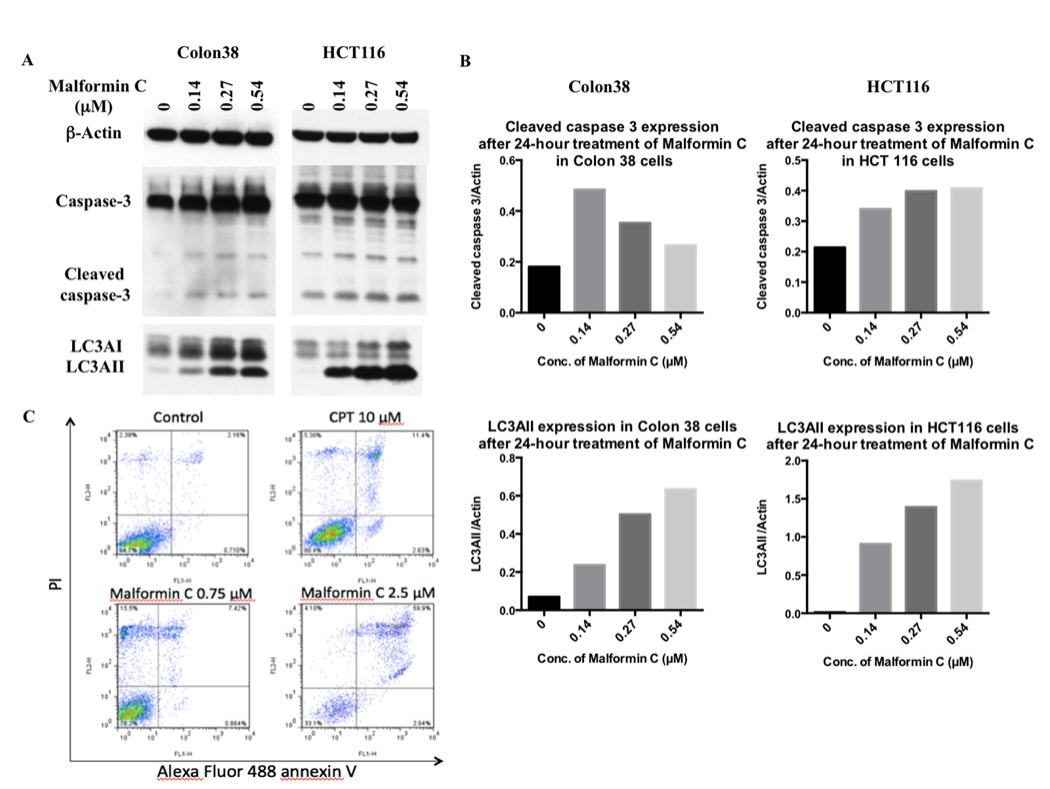
Expression of CASPASE 3 and LC3 and apoptosis assay in cells treated with Malformin C. **(A, B)** The expression of cleaved CASPASE 3, total CASPASE 3, LC3AI and LC3AII in Colon 38 and HCT 116 cells treated with different concentrations of Malformin C (0μM, 0.14μM, 0.27μM, 0.54μM for 24 hours was tested by Western blot, with β-Actin expression as an internal control. During autophagy process, LC3AI will be converted into LC3AII to be functional, so we analyzed LC3AII expression for autophagy process. **(C)** Malformin C led to late apoptosis and necrosis after 24-hour treatment examined by apoptosis assay. Untreated or Malformin C-treated Colon 38 cells (2×10^6^) were stained with Alexa Fluor 488 annexin V and PI and processed by flow cytometer. The percentage of early apoptotic population (lower right panel), late apoptotic population (upper right panel) and necrotic population (upper left panels) is shown in the graph. CPT, a known apoptosis-inducing agent, was used as a control. Three independent experiments were performed with duplicates for each condition, and one typical result is shown here.

The expression of CASPASE 3 and LC3 was also examined by Western blot. During autophagy, LC3AI is converted to LC3AII through lipidation, and LC3AII serves as an indicator of autophagy. So we analyzed LC3AII expression to test Malformin C’s effect on autophagy. After 24-hour treatment of Malformin C, we observed a dose-dependent up-regulation of cleaved CASPASE 3 in HCT 116 cells, and a dose-dependent up-regulation of LC3AII in both Colon 38 and HCT 116 cells (Fig 4A and 4B). But there was no dose-response for the increased expression of cleaved CASPASE 3 in Colon 38 cells, and it could due to the fact that some apoptotic cells detached from the plate during culture and were lost when the media was removed. No significant changes of cleaved CASPASE 3 and LC3AII expression were detected after 4-hour and 8-hour treatment (S5 Fig). To further investigate the death process of those DNA-damaged cells, we conducted the apoptosis assay and found a significantly larger group of late apoptotic cells and necrotic cells after Malformin C exposure, while the percentage of early apoptotic cells showed no difference from the control (Fig 3E). All in all, we found that 24-hour treatment of Malformin C could cause DNA damage and lead to cell death through apoptosis, autophapy and necrosis.

### Pathological investigation on Malformin C treated mice

**Fig 5:**
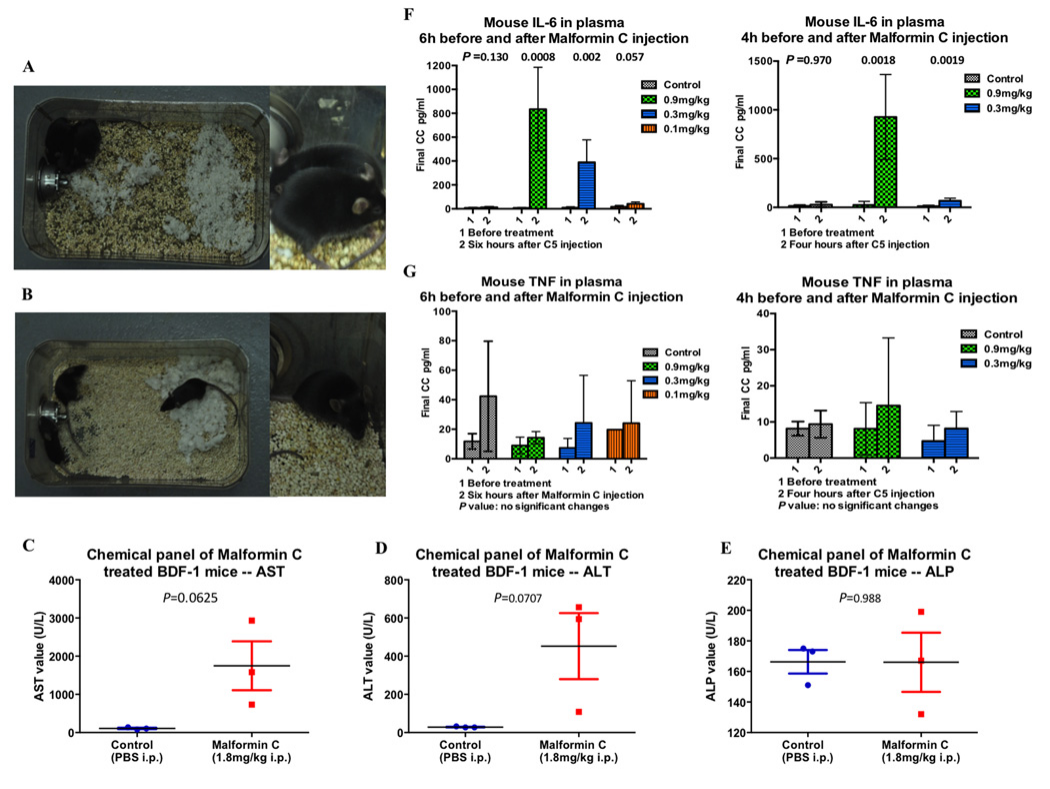
Pathological investigation of Malformin C treated mice. **(A)** Observation of mice prior to death injected with 1.8mg/kg Malformin C. The mice exhibited hunched posture, huddling and piloerection. **(B)** Control mice injected with PBS were active, with normal haircoats. **(C)** The parameter indicating the most different between treated and control mice was AST (aspartate aminotransferase). AST elevation may be lower than reported due to a hemolysis 3+, but is still abnormal. **(D)** There were also mild differences in ALT (alanine amino transferase) values between treated and control mice. **(E)** ALP (alkaline phosphatase) could be elevated with cholestasis, but in this case, the average value for the treated and control mice was identical, and thus not considered to be a significant finding. **(F)** Mouse IL-6 level in the plasma of Colon 38 bearing BDF-1 mice. The left graph showed a dose dependent increase of mouse IL-6 in the plasma 6 hours after 0.9mg/kg, 0.3mg/kg and 0.1mg/kg Malformin C injection compared to PBS group, while the right graph showed similar effects 4 hours after 0.9mg/kg, 0.3mg/kg Malformin C injection. **(G)** Mouse TNF level in the plasma of Colon 38 bearing BDF-1 mice. No significant changes were shown either 6 hours or 4 hours before and after different concentrations of Malformin C injection compared to PBS injection group.

In order to study the toxicity of Malformin C itself, six 9±0.5 week old BDF-1 mice were randomly assigned to control group and 1.8mg/kg Malformin C group with three mice in each. Prior to death (about 6 hours after injection), all three mice in the treated cage exhibited hunched posture, huddling, reluctance to move and piloerection (Fig 5A). Conversely, all three mice in the control group were active with a smooth haircoat (Fig 5B). No significant gross lesions were noted in any of the six mice upon post-mortem examination. Clinical chemistry panels on the treated mice indicated marked elevation in AST and a mild increase in ALT (Fig 5C and 5D, S2 Table), consistent with acute hepatocellular damage. Histologically, all the tissues were stained and the only finding in the treated mice was in the liver and consisted of Kupffer cell activation, mild infiltration by neutrophils and occasional hepatocellular apoptosis. While these changes are suggestive of and consistent with that expected in an acute inflammatory response, they are subtle. Clinical signs prior to death did not support a cardiac or neurologic phenotype and there were no gross or histologic lesions within the heart or brain of any of these mice. Culture of the abdominal cavity and spleen of the three treated mice, as well as one control mouse, did not yield bacterial growth. The small micro-granulomas noted in all mice are considered to be a chronic lesion and not associated with the given compound. Additionally, data from the following study of Colon 38 bearing mice showed a noted dose-dependent elevation of IL-6 in Malformin C injected groups, which also indicates the existence of acute inflammation cause by Malformin C (Fig 5F). In this study, significant increase of TNF in tumor-bearing model was not observed (Fig 5G).

### Malformin C’s anti-cancer effects and its in vivo toxicity

**Fig 6:**
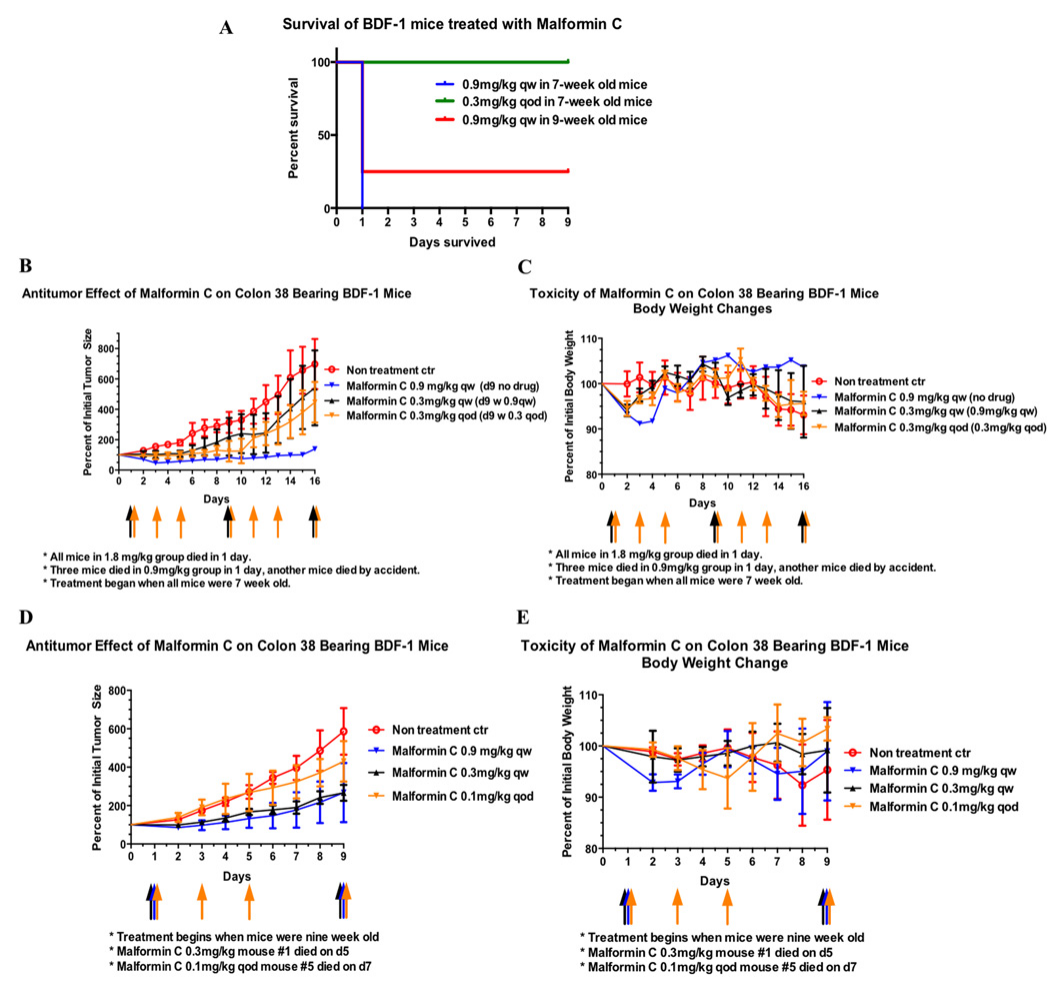
The anti-cancer effects and in vivo toxicity of Malformin C. **(A)** Survival of BDF-1 mice treated with Malformin C. All the mice in 2.6mg/kg and 1.8mg/kg group died within 1 day after injection, and all the mice in 0.3mg/kg and 0.1mg/kg survived throughout the experiment. There were 5 mice in each group and the week age indicated was that at the beginning of the experiment. One of the mice in 0.9mg/kg qw 7-week old group died by accident and was therefore excluded from the graph. Malformin C was given by i.p., qw means every week, qod means every other day. **(B)** Tumor growth of Colon 38 bearing BDF-1 mice treated with Malformin C. All the mice were 7 weeks old on Day 0. Control group was treated with 0.1ml/10g PBS (the intake volume was the same for all groups), 0.9mg/kg group was injected with Malformin C on Day 1, 0.3mg/kg q.w. group was treated with 0.3mg/kg on Day 1 and treated with 0.9mg/kg on Day 9 in order to confirm age and toxicity effect, 0.3mg/kg q.o.d. group was treated with 0.3mg/kg Malformin C on Day 1, 3, 5, 9, 11, 13 and 16. **(C)** Body weight changes of corresponding groups in A were measured. **(D)** Tumor growth of Colon 38 bearing BDF-1 mice treated with Malformin C. All the mice were 8 weeks old on Day 0. Control group was treated with 0.1ml/10g PBS, 0.9mg/kg group was injected with Malformin C on Day 1 and 9, 0.3mg/kg q.w. group was treated with 0.3mg/kg on Day 1 and 9, 0.1mg/kg q.o.d. group was treated with 0.1mg/kg Malformin C on Day 1, 3, 5 and 9. **(E)** Body weight changes of corresponding groups in C were measured.

Malformin C was reported to be toxic in vivo 0.9mg/kg for newborn rats, but original data weren’t available nor were there any follow-up studies. In our preliminary study, Malformin C was delivered i.p., and all the mice (both 7 and 9 weeks old when treated) in 1.8mg/kg and 2.6mg/kg groups died. For 7-week old mice, three out of four of the mice in 0.9mg/ml group died within 24 hours (Fig 6A), while there was no mouse death in 0.9mg/ml group when they were treated at 9 weeks old (Fig 6D). All the mice deaths were unexpected and caused by the acute toxicity of Malformin C. In order to confirm that the cause of death was age related, we conducted the second injection when the mice were 8 weeks old and 9 weeks old, using the mice that were originally given 0.3mg/kg qw (Fig 6B). No mouse death was observed this time. Again, 9-week old mice didn’t die in Malformin C 0.9mg/ml group in our following study (Fig 6D). Therefore, for 9-week old mice 0.9mg/kg was their highest dosage limit for treatment, the cause of death at 0.9mg/kg might be related to mouse maturity, and significant acute toxicity occurred within 24 hours in mice treated with Malformin C and there was no obvious accumulating effect since all the mice in 0.3mg/kg q.o.d. group survived.

The anti-tumor effect of Malformin C was measured by the volume of transplanted Colon 38 tumor in BDF-1 mice, while body weight loss was recorded as a primary indicator of side effects. As we could see from Fig 5B, the tumor of the only 7-week old mouse which survived 0.9mg/kg Malformin C injection did not grow until Day 15. But when treated at 9 weeks old, the tumors in 0.9mg/kg group grew up to 2.7-fold of the original size on Day 9 (Fig 6D). There was no significant difference between 0.3mg/kg q.w. and 0.3mg/kg q.o.d.(Fig 6B), and no significant difference between 0.9mg/kg q.w. and 0.3mg/kg q.w. (Fig 6D). Therefore, we suggest that Malformin C’s anti-tumor effect might be related to its age and in vivo toxicity, and 0.3mg/kg q.w. was the best therapeutic dosage. As for the body weight changes of Colon 38 bearing BDF-1 mice (Fig 6C and 6E), there was up to 9% weight loss one or two days after Malformin C injection, and the growth of tumor was the major reason for weight loss aside from Malformin C’s toxicity. Also we found that the weight loss in 0.9mg/kg group was more obvious than that in 0.3mg/kg group, while it took a longer time for mice in 0.9mg/kg group to recover as well.

## Discussion

A good anti-cancer drug needs to be effective against malignant tumors yet tolerable to cancer patients. Multidrug resistance is one major obstacle to the anti-tumor effect. P-glycoprotein (P-gp), also known as ATP-binding cassette sub-family B member 1 (ABCB1), is an ATP-dependent drug efflux pump for xenobiotic compounds which often leads to multi-drug resistance to anti-cancer drugs. It is extensively distributed and expressed in the intestinal epithelium, hepatocytes, renal proximal tubular cells, adrenal gland and capillary endothelial cells comprising the blood-brain and blood-testis barrier. According to our study, Malformin C is highly resistant to P-gp over-expressed cell lines and this resistance is further confirmed by the reversal effect of P-gp antagonist. This means that, first of all, Malformin C’s effect will be compromised if taken orally since there is vast MDR protein in GI tract. Therefore, we choose intra-peritoneal injection for in vivo study. Secondly, acute toxicity in vivo is less likely due to Central Nervous System (CNS) damage because there is also abundant MDR protein in the blood-brain barrier. However, Malformin C is not resistant to all drug-resistant cell lines. It is actually more sensitive to CPT resistant cell line, which suggests combined Malformin C and CPT-11 for colon cancer research.

Our experiments indicate that Malformin C exhibits a potent cell growth inhibition against colon cancer cell lines possibly by inducing DNA damage, phosphorylating H2A.X, perturbing cell cycle progression and consequently leading to multiple forms of cell death. A transcription factor and tumor suppressor protein, p53, is a sensor of DNA damage, and plays a pivotal role in growth arrest and apoptosis. The activation of p53 can trigger cell cycle arrest both in G1 and G2/M phases, and traditionally many DNA-damage-inducing therapeutic drugs, including CPT, target tumors via p53-mediated apoptosis [34–36]. As is presented here, Malformin C up-regulates the expression of p53 and results in G2/M arrest in Colon 38 cells at 0.5–0.8μM. Furthermore, 24-hour treatment with Malformin C induces multiple forms of cell death through apoptosis, autophagy and necrosis in different colon cancer cell lines according to immunofluorescence micrographs, Western blot and apoptosis assay. In addition, Malformin C is reported to inhibit Bleomycin-induced G2 checkpoint in Jurkat cells with an IC_50_ of 0.9nM [14]. However, no similar G2 checkpoint inhibition is shown for SN38-induced G2/M arrest when treated with low concentration of Malformin C in Colon 38 cells. The difference may be because Jurkat cells lack functional p53 and could only repair DNA in G2 checkpoint but Colon 38 cells can repair in both G1 and G2 checkpoints.

Malformin C is claimed to have anti-cancer activity in recent publications, but this is the first time that Malformin C’s in vivo anti-cancer effects have been reported. We carried out a series of animal studies to investigate the lethal dosage and therapeutic dosage, anti-tumor effect and toxicity, as well as age effects. Firstly, Malformin C injection of 0.1mg/kg every other day has no anti-tumor effect yet has a high resistant risk, whereas 0.9mg/kg per week Malformin C either causes fatal toxicity in 7-week old mice or has no advantage compared to 0.3mg/kg per week dosage in 9-week old mice. Therefore, Malformin C injection at 0.3mg/kg per week is the best therapeutic dosage, at which tumor size is effectively inhibited yet toxicity is well tolerated. The therapeutic index of Malformin C, however, is too low to be an anti-cancer drug. Considering this, new analogs of Malformin C will be synthesized and analyzed in the hope of separating its toxicity and anti-tumor effect. Secondly, age plays an important role in this study. This phenomenon is clearly illustrated in 0.9mg/kg per week Malformin C group in that the anti-tumor effect and in vivo toxicity of this compound is notably dependent on the week-age of the mice on treatment day. This implies the microenvironment inside the mouse especially those related to maturity, instead of the tumor factor itself, is critical for Malformin C’s function. Additionally, Malformin C is a two-edged weapon for body weight changes. On one hand, it restricts the tumor size and to some extent prevents weight loss from cancer cachexia. On the other hand, its acute toxicity also causes weight loss. Finally, an initial pathological experiment helps us to look into the reason for the acute toxicity of Malformin C. An acute yet subtle inflammatory response has been proven after Malformin C administration. Evidence does not support a cardiac or cerebral cause of death. In future study, lower dosage of the Malformin C will be tested to allow more time for lesion development, in order to further investigate its toxicology.

In summary, we report that Malformin C, a toxic fungal cyclic pentapeptide, is active against different cancer cells including Colon 38 in vitro through multiple mechanisms. However, its *in vivo* anti-tumor effects is largely restricted by the acute lethal toxicity, which is closely related to the maturity of the mice. In our future work, we will synthesize new analogs of Malformin C to explore the structure activity relationship, as well as to investigate their effects on the tumor and the microenvironment inside the body. The hematological effects and cytokine changes caused by this unique compound will also be addressed in order to tap the potential of clinical use in cancer treatment.

## Supporting Information

S1 ARRIVE Checklist NC3Rs ARRIVE guidelines checklist for Malformin C experiments. (PDF)

S1 Fig Cell cycle analysis of Colon 38 cells exposed to Malformin C for different time intervals. Cell cycle analysis of Colon 38 cells exposed to 0.54μM Malformin C for 15 hours, 24 hours, 30 hours and 48 hours. No time-dependence was shown for the G2-M arrest of Colon 38 cells induced by Malformin C.
(TIF)

S2 Fig Cell cycle analysis of HCT 116 cells treated by Malformin C and its combinations. **(A)** Cell cycle progression of HCT 116 cells exposed to an increasing concentration of Malformin C for 24 hours **(B)** Cell cycle progression of HCT 116 cells exposed to an increasing concentration of Malformin C for 48 hours. There was no significant change of cell cycle progression in HCT 116 cells treated by Malformin C at the concentration of 90nM, 270nM and 810nM. **(C)** Cell cycle analysis of HCT 116 cells treated with combinations of Malformin C and SN38. All the cells were exposed to respective compounds indicated above the graph for 24 hours and the analysis was done in duplicate. When treated with SN38, no significant changes of cell cycle progression were observed without or with different dosage of Malformin C.
(TIF)

S3 Fig Expression of phosphorylated H2A.X in Colon 38 and HCT 116 cells treated with Malformin C. The expression of phosphorylated H2A.X in Colon 38 and HCT 116 cells treated with different concentrations of Malformin C (0μM, 0.14μM, 0.27μM, 0.54μM) and Hydroxyurea (1mM, 2mM) for 2-hour, 4-hour, 8-hour and 24-hour tested by Western blot, with β-Actin expression as an internal control. BenchMark^TM^ Pre-stained Protein Ladder was used in this experiment.
(TIF)

S4 Fig Expression of total H2A.X in Colon 38 and HCT 116 cells treated with Malformin C. The expression of total H2A.X in Colon 38 and HCT 116 cells treated with different concentrations of Malformin C (0μM, 0.14μM, 0.27μM, 0.54μM) and Hydroxyurea (1mM, 2mM) for 2-hour, 4-hour, 8-hour and 24-hour tested by Western blot, with β-Actin expression as an internal control. BenchMark^TM^ Pre-stained Protein Ladder was used in this experiment.
(TIF)

S5 Fig Expression of cleaved CASPASE 3 and LC3AII in Colon 38 and HCT 116 cells treated with Malformin C. The expression of cleaved CASPASE 3 and LC3AII in Colon 38 and HCT 116 cells treated with different concentrations of Malformin C (0μM, 0.14μM, 0.27μM, 0.54μM) for 4-hour, 8-hour and 24-hour was tested by Western blot, with β-Actin expression as an internal control. BenchMark^TM^ Pre-stained Protein Ladder was used in this experiment.
(TIF)

S1 File Detailed results of pathological study for Malformin C. (DOC)

S1 Table Growth inhibition of Malformin C for different cell lines. (PDF)

S2 Table Chemical panels of Malformin C treated BDF-1 mice. (PDF)
