# Supplementary material for: Study of Malformin C, a Fungal Source Cyclic Pentapeptide, as an Anti-Cancer Drug
Source: PLoS One. 2015 Nov 5;10(11):e0140069. doi: 10.1371/journal.pone.0140069 (PMC4635020; doi:10.1371/journal.pone.0140069)
Supplement: S1 File — (DOC) [file pone.0140069.s007.doc]

**Case number: N1214714**

**Species:** *Mus musculus*

**Breed/Strain:** B6D2F1 (BDF-1)

**Room:** BCMM B19

**PI:** Yung-Chi Cheng

**Contact:** Jing Wang

**Pathologist:** Amanda Beck

**Senior Pathologist:** Caroline Zeiss

**History**

Toxicity study. Treatment group = 1.8mg/kg Malformin C. Control group = PBS.

**Clinical Chemistry**

*See attached for complete report

Overall, the clinical chemistry panel indicates acute hepatocellular damage. The parameter indicating the most different between treated and control mice was AST (aspartate aminotransferase). AST is an indicator of liver and/or muscle injury and will be elevated with such insults. AST can also be artifactually elevated with extensive sample hemolysis, as it is also present in erythrocytes. Prominent hemolysis was noted in the samples from treated Mouse 2 and Mouse 3, but not Mouse 1. However, the disclaimer provided with the report indicates that “AST may be lower than reported due to a hemolysis 3+, but is still abnormal.”

There were also mild differences in ALT (alanine amino transferase) values between treated and control mice. ALT elevations are fairy specific for hepatic injury, though it is also found in skeletal muscle.

As for other parameters that showed deviation from the normal range, ALP (alkaline phosphatase) can be elevated with cholestasis, but in this case, the average value for the treated and control mice is identical, and thus, this is not considered to be a significant finding. Elevation in potassium across all mice (treated and control) is due to antemortem respiratory acidosis that occurs during euthanasia by carbon dioxide, and is thus an artifactual finding (Traslavina RP et al 2010). Elevation in glucose in all of the mice except one can be attributed to stress due to handling, injection and manipulation. Elevation in phosphorus across all mice (treated and control) may be attributed to hemolysis of the sample and is also considered artifactual.

Other values were only minimally out of the normal range and are not considered to be significant.

|  | **Treated 1** | **Treated 2** | **Treated 3** | **Treated (Avg)** | **Control 1** | **Control 2** | **Control 3** | **Control (Avg)** |
| --- | --- | --- | --- | --- | --- | --- | --- | --- |
| **AST (U/L)** | 732 | 1583 | 2930 | 1748 | 139 | 106 | 81 | 109 |
| **ALT**  **(U/L)** | 108 | 656 | 594 | 453 | 32 | 27 | 27 | 29 |
| **ALP**  **(U/L)** | 132 | 167 | 199 | 166 | 175 | 173 | 151 | 166 |

**Bacteriology (M1231628 – M1231634)**

Culture of the abdominal cavity and spleen from the three treated mice and one control mouse did not yield any growth.

**Gross findings:**

Prior to death, all three mice in the treated cage exhibited hunched posture, huddling, reluctance to move and piloerection (Fig 1). Conversely, all three mice in the control group were active with a smooth haircoat (Fig 2).

No significant gross lesions were noted in any of the six mice at post-mortem examination.

**Histopathologic findings**

Mouse #1 (Treated #1)

The following organs are examined histologically:

Slide A: Head (brain, pituitary gland)

Slide B: Heart, lungs, thymus

Slide C: Liver

Slide D: Spleen, kidneys, salivary glands, submandibular lymph node, pancreas, adrenal glands

Slide E: Stomach, small intestine, large intestine

Slide F: Uterus, ovaries, urinary bladder

Slide G: Hindlimb, sternum

All organs are within normal limits histologically, unless described below.

Liver: Diffusely, hepatocytes are mildly to moderately swollen with wispy to granular eosinophilic cytoplasm (glycogenosis). Diffusely, Kupffer cells are plump and active. Multifocally, there are occasional small microgranulomas composed of lymphocytes and histiocytes admixed with occasional neutrophils.

Spleen: Multifocally, there is mild expansion of lymphoid follicles with occasional coalescence.

Mouse #2 (Treated #2)

The following organs are examined histologically:

Slide A: Head (brain, pituitary gland)

Slide B: Heart, lungs, thymus, trachea, esophagus

Slide C: Liver

Slide D: Spleen, pancreas, kidneys, salivary glands, submandibular lymph node, adrenal gland

Slide E: Stomach, small intestine, large intestine

Slide F: Uterus, ovaries, urinary bladder

Slide G: Hindlimb, sternum

All organs are within normal limits histologically, unless described below.

Liver: Diffusely, hepatocytes are mildly to moderately swollen with wispy to granular eosinophilic cytoplasm (glycogenosis) and occasional hepatocytes are markedly swollen. There is rare single cell hepatocellular apoptosis. Diffusely, Kupffer cells are plump and active. Multifocally, there are occasional small microgranulomas composed of lymphocytes and histiocytes admixed with occasional neutrophils and small numbers of neutrophils are seen within sinusoids.

Mouse #3 (Treated #3)

The following organs are examined histologically:

Slide A: Head (brain, pituitary gland)

Slide B: Heart, lungs, thymus, trachea, esophagus

Slide C: Liver

Slide D: Spleen, kidneys, salivary glands, submandibular lymph node, adrenal glands

Slide E: Stomach, small intestine, large intestine, pancreas

Slide F: Uterus, ovaries, urinary bladder

Slide G: Hindlimb, sternum

All organs are within normal limits histologically, unless described below.

Liver: Diffusely, hepatocytes are mildly to moderately swollen with wispy to granular eosinophilic cytoplasm (glycogenosis) and occasional hepatocytes are markedly swollen. There is rare single cell hepatocellular apoptosis. Diffusely, Kupffer cells are plump and active. Multifocally, there are occasional small microgranulomas composed of lymphocytes and histiocytes admixed with occasional neutrophils and small numbers of neutrophils are seen within sinusoids.

Mouse #4 (Control #1)

The following organs are examined histologically:

Slide A: Head (brain, pituitary gland)

Slide B: Heart, lungs, thymus, trachea, esophagus

Slide C: Liver

Slide D: Spleen, pancreas, kidneys, salivary glands, submandibular lymph node, adrenal gland

Slide E: Stomach, small intestine, large intestine

Slide F: Uterus, ovaries

Slide G: Hindlimb, sternum

All organs are within normal limits histologically, unless described below.

Liver: Diffusely, hepatocytes are mildly to moderately swollen with wispy to granular eosinophilic cytoplasm (glycogenosis). Multifocally, there are occasional small microgranulomas composed of lymphocytes and histiocytes.

Mouse #5 (Control #2)

The following organs are examined histologically:

Slide A: Head (brain, pituitary gland)

Slide B: Heart, lungs, thymus

Slide C: Liver

Slide D: Spleen, kidneys, salivary glands, submandibular lymph node, adrenal gland

Slide E: Stomach, small intestine, large intestine

Slide F: Uterus, ovaries, urinary bladder

Slide G: Hindlimb, sternum

All organs are within normal limits histologically, unless described below.

Liver: Diffusely, hepatocytes are mildly to moderately swollen with wispy to granular eosinophilic cytoplasm (glycogenosis). Multifocally, there are occasional small microgranulomas composed of lymphocytes and histiocytes.

Mouse #6 (Control #3)

The following organs are examined histologically:

Slide A: Head (brain, pituitary gland)

Slide B: Heart, lungs, thymus, esophagus

Slide C: Liver

Slide D: Spleen, pancreas, kidneys, salivary glands, submandibular lymph node, adrenal gland

Slide E: Stomach, small intestine, large intestine

Slide F: Uterus, ovaries, urinary bladder

Slide G: Hindlimb, sternum

All organs are within normal limits histologically, unless described below.

Liver: Diffusely, hepatocytes are mildly to moderately swollen with wispy to granular eosinophilic cytoplasm (glycogenosis). Multifocally, occasional Kupffer cells are plump and active and there are small microgranulomas composed of lymphocytes and histiocytes.

**Morphologic Diagnoses**

Mouse #1 (Treated #1)

1. Liver; diffuse Kupffer cell activation and mild multifocal neutrophilic infiltration

Mouse #2 (Treated #2)

1. Liver; diffuse Kupffer cell activation, mild multifocal neutrophilic infiltration and rare single cell apoptosis

Mouse #3 (Treated #3)

1. Liver; diffuse Kupffer cell activation, mild multifocal neutrophilic infiltration and rare single cell apoptosis

**Comment:**

Clinical chemistry panels on the treated mice indicated marked elevation in AST and a mild increase in ALT, consistent with acute hepatocellular damage. Histologically, the only finding in the treated mice was in the liver and consisted of Kupffer cell activation, mild infiltration by neutrophils and occasional hepatocellular apoptosis. While these changes are suggestive of and consistent with that expected in an acute inflammatory response, they are subtle. It is recommended that a lower dosage of the compound is tested in the future so that mice can survive for a longer period of time, allowing more time for lesion development.

Additionally, because it is possible that the tested compound induces an acute phase response and subsequent cytokine storm, analysis of serum TNFa levels are recommended. Clinical signs prior to death did not support a cardiac or neurologic phenotype and there were no gross or histologic lesions within the heart or brain of any mouse.

Culture of the abdominal cavity and spleen of the three treated mice, as well as one control mouse, did not yield bacterial growth. The small microgranulomas noted in all mice are considered to be a chronic lesion and not associated with the given compound.


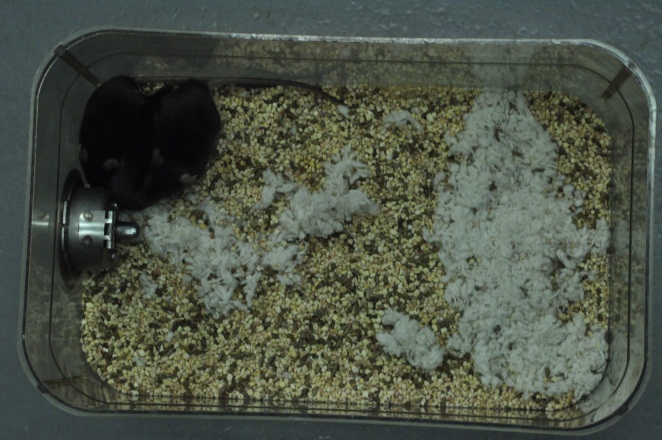

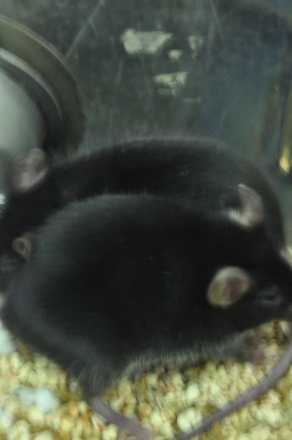


**Figure 1.** Treated mice exhibit hunched posture, huddling and piloerection.


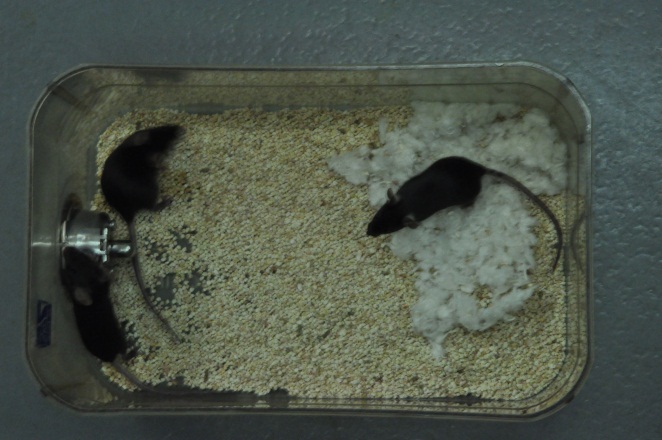

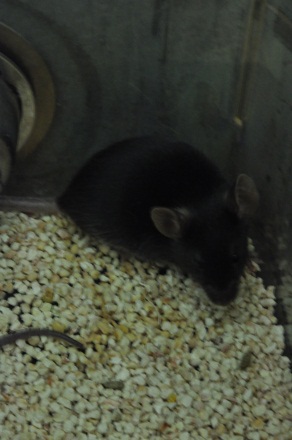


**Figure 2**. Control mice are active, with normal haircoats.
