# Supplementary material for: Study of Malformin C, a Fungal Source Cyclic Pentapeptide, as an Anti-Cancer Drug
Source: PLoS One. 2015 Nov 5;10(11):e0140069. doi: 10.1371/journal.pone.0140069 (PMC4635020; doi:10.1371/journal.pone.0140069)
Supplement: S1 Table — (PDF) [file pone.0140069.s008.pdf]

Supplementary Table 1 Growth inhibition of Malformin C for different cell lines

Cell Lines	IC_{50} (μM)[a]				
	Colon 38	HCT 116	PanO2	H1975	CEM
Malformin C [b]	0.27±0.07	0.18±0.02	0.29±0.05	0.16±0.04	0.030±0.008
L-OddC	1.1±0.08	0.88±0.1	5.2±0.7	0.34±0.04	0.026±0.003

[a] Values were means ± SD from more than three independent experiments, with each data point done in duplicate, and all the cells were exposed to different drugs for three generations.

[b] There was significant difference of Malformin C's IC_{50} among different cell lines according to one-way ANOVA statistical analysis ($P<0.01$); and the IC_{50} of Malformin C was significantly different from that of L-OddC in Colon 38 cells, HCT 116 cells and PanO2 cells ($P<0.01$) by two-sided t test.
