# Supplementary material for: Study of Malformin C, a Fungal Source Cyclic Pentapeptide, as an Anti-Cancer Drug
Source: PLoS One. 2015 Nov 5;10(11):e0140069. doi: 10.1371/journal.pone.0140069 (PMC4635020; doi:10.1371/journal.pone.0140069)
Supplement: S2 Table — (PDF) [file pone.0140069.s009.pdf]

Supplementary Table 2 Chemical panels of Malformin C treated BDF-1 mice

Chemical panels (U/L)	Malformin C group (1.8mg/kg i.p.)				Control group (PBS i.p.)			
	1	2	3	Average	1	2	3	Average
AST[a]	732	1583	2930	1748	139	106	81	109
ALT[b]	108	656	594	453	32	27	27	29
ALP[c]	132	167	199	166	175	173	151	166

Note: a. The parameter indicating the most different between treated and control mice was AST (aspartate aminotransferase). AST elevation may be lower than reported due to a hemolysis 3+, but is still abnormal. **b.** There were also mild differences in ALT (alanine amino transferase) values between treated and control mice. **c.** ALP (alkaline phosphatase) could be elevated with cholestasis, but in this case, the average value for the treated and control mice was identical, and thus not considered to be a significant finding.
